# Video vs. direct laryngoscopy for tracheal intubation in neonates: a meta-analysis

**DOI:** 10.3389/fped.2025.1674255

**Published:** 2025-10-23

**Authors:** Xiang Li, Xulin Zhang, Dongxu Chen, Chao Yu, Xiaoqin Jiang

**Affiliations:** ^1^ Department of Anesthesiology, West China Second University Hospital, Sichuan University, Chengdu, China; ^2^ Key Laboratory of Birth Defects and Related Diseases of Women and Children, Ministry of Education, Sichuan University, Chengdu, China; ^3^ Department of Anesthesiology, Chengdu Hi-Tech Zone Hospital for Women and Children, Chengdu, China

**Keywords:** neonate, endotracheal intubation, videolaryngoscopy, direct laryngoscopy, trial sequential analysis

## Abstract

**Purpose:**

This study aimed to synthesize data from randomized controlled trials (RCTs) evaluating the effectiveness and safety of videolaryngoscopy vs. direct laryngoscopy in neonates undergoing endotracheal intubation.

**Methods:**

This meta-analysis was conducted on June 1, 2024, in MEDLINE, Embase, Cochrane Central, and CINAHL EbscoHost databases to identify relevant trials. Primary outcome was the success rate of intubation on the first attempt. Secondary outcomes included the time required for successful intubation, number of intubation attempts, adverse events related to both non-airway and airway complications. Trial sequential analysis (TSA) was performed to rule out the possibility of false positive result.

**Results:**

Nine RCTs involving 1,059 neonates were included. Videolaryngoscopy significantly improved the success rate of first-attempt intubation [risk ratio (RR) 1.21, 95% CI 1.06–1.38], TSA conﬁrmed these findings. Subgroup analyses indicated that videolaryngoscopy was particularly beneficial for inexperienced clinicians or when used in the neonatal intensive care unit. However, videolaryngoscopy did not significantly reduce the number of intubation attempts [mean difference (MD) −0.22, 95% CI −0.44–0.01] and had a similar time to successful intubation as direct laryngoscopy (MD 0.91, 95% CI −0.32–2.14). Videolaryngoscopy was associated with less airway trauma (RR 0.23, 95% CI 0.06–0.89). Additionally, videolaryngoscopy showed minimal differences in the occurrence of bradycardia, desaturation, or low oxygen saturation levels during intubation.

**Conclusion:**

The current evidence suggested that videolaryngoscopy enhanced the success rate of first-attempt intubation and reduced airway trauma, while requiring a similar time required for successful intubation compared to direct laryngoscopy.

**Systematic Review Registration:**

https://www.crd.york.ac.uk/PROSPERO/view/CRD42024552392, PROSPERO CRD42024552392.

## Introduction

Endotracheal intubation is a critical life-saving technique employed in various clinical scenarios for neonates. The success of this procedure, crucial for both experienced practitioners and those in training, depends heavily on clear visualization of the airway and its associated structures. Neonatal airway anatomy presents unique challenges compared to adults, including a smaller mouth and airway size, the keyhole appearance of the glottis, a disproportionately large tongue, epiglottis, and arytenoids, as well as extensive secretions (1). A retrospective study of 7,708 neonatal intubations found that 1,474 (22%) required three or more attempts (2), and such repeated attempts were linked with a 4–10-fold increase in the risk of adverse events (2, 3).

The use of advanced equipment can decrease the time required for intubation and improve the success rate on the first attempt (4). Extensive trials have demonstrated that videolaryngoscopy significantly improves the first-attempt success rate during elective intubations in adults (5) and children (6), as well as in emergency scenarios (7). However, randomized controlled trials (RCTs) in neonates have yielded mixed results regarding the efficacy of video laryngoscopes, with studies indicating either a positive impact or no effect at al. (8, 9). Additionally, a multicenter retrospective study indicated that videolaryngoscopy did not improve the success of first-attempt intubations (10).

Previous systematic reviews have emphasized the need for well-designed, adequately powered RCTs to confirm the effectiveness and safety of videolaryngoscopy in neonatal intubation (11). Recently, new RCT evidence has suggested that videolaryngoscopy may indeed lead to a higher number of successful first-attempt intubations compared to direct laryngoscopy (12). With this background, our objective was to summarize the current evidence from RCTs to determine if videolaryngoscope can increase success rates in neonatal endotracheal intubation.

## Materials and methods

This study's protocol has not been previously published. The manuscript is prepared in accordance with the PRISMA (Preferred Reporting Items for Systematic reviews and Meta Analyses) statement (13). The study was registered in the PROSPERO database (CRD42024552392).

### Eligibility criteria

Inclusion and exclusion criteria were predefined based on the PICOS framework:

***Patients:*** Neonates requiring intubation in the delivery room, operating room, or neonatal intensive care unit (NICU). Studies involving older individuals were included if neonatal data could be extracted independently.
***Intervention:*** Videolaryngoscopy using any device suited for neonatal orotracheal intubation, such as the Pentax Airway Scope, GlideScope, Neoview, Airtraq, C-MAC, and Truview.
***Comparator:*** Conventional direct laryngoscopy, such as the HEINE, RUSCH
***Outcomes:*** The primary outcome was the success rate at the first attempt. Secondary outcomes included:
  a. Time required for successful intubation, quantified from the initial insertion of the laryngoscope blade into the mouth to the final confirmation of endotracheal tube (ETT) placement by clinical exam, increased peripheral oxygen saturation (SpO_2_), detection of end-tidal carbon dioxide (EtCO_2_), or chest radiograph.
  b. Number of intubation attempts, with each insertion and removal of the laryngoscope blade counted as one attempt, regardless of success.
  c. Non-airway-related adverse effects such as episodes of bradycardia, desaturation, or the lowest recorded SpO_2_ from the start of intubation until normalization (SpO_2_ > 95%).
  d. Airway-related adverse effects including trauma to oral, pharyngeal, and laryngeal structures, such as lacerations and perforations, assessed through visual or laryngoscopic examination. No outcomes required follow-up post-intubation.
***Types of studies:*** Only RCT were included.
***Exclusion criteria:*** Studies were excluded if neonatal data could not be separately extracted.

### Information sources and search

A systematic search was conducted on June 1, 2024, in MEDLINE, Embase, Cochrane Central, and CINAHL EbscoHost databases to identify relevant trials. The search strategies are detailed in Supplement 1. Reference lists of included studies were also reviewed to find additional eligible articles.

Identified articles were managed using EndNote v20.0 (Clarivate Analytics) to remove duplicates. Two investigators (XL and XLZ) independently assessed the abstracts for study eligibility based on the criteria specified above, followed by full-text screening. Only full reports of the studies were included in this review. Any disagreements regarding a trial's eligibility were resolved through mutual discussion. We meticulously recorded the selection process to ensure sufficient detail for completing a PRISMA flow diagram.

### Data extraction

Data extraction was performed by two authors (DXC and XL) from the reports deemed eligible. For each included study, information was collected on the method of randomization, blinding, intervention, stratification, and whether the trials were conducted at a single or multiple centers. Only data pertaining to our predefined primary and secondary outcomes, presented as either event counts or as means and standard deviations, were included in our analysis.

### Risk of bias in individual studies

We employed the Cochrane Risk of Bias tool (14) to evaluate the methodological quality of the included studies, with two authors (DXC and XL) conducting the analysis independently. This tool assesses the potential for selection bias (via random sequence generation and allocation concealment), performance bias (through blinding of participants and personnel), detection bias (by blinding of outcome assessors), and attrition bias. In instances of disagreement between the two authors (DXC and XL), a third author (XQJ) was consulted to mediate and resolve any discrepancies. A trial was deemed to be at low risk of bias if it demonstrated adequate procedures for random sequence generation, allocation concealment, and blinded outcome assessment.

### Certainty of evidence across trials

The overall certainty of evidence across pooled outcomes was also assessed using the Grading of Recommendation, Assessment, Development, and Evaluation (GRADE) guidelines (15). Two authors (XLZ and LH) independently assessed the included trials for the certainty of evidence using established criteria. The degree of bias identified was then used to categorize the quality of the overall pooled outcomes, which ranged from high to very low.

### Statistical analysis

For dichotomous data, we reported results using risk ratios (RR) with 95% confidence intervals (CIs). For continuous data, the mean difference (MD) was employed when outcomes were measured consistently across trials. In cases where trials measured the same outcome using different methods, we utilized the standardized mean difference (SMD). When trials reported continuous data as medians and interquartile ranges (IQR), and these data passed the skewness test, we converted medians to means and estimated the standard deviation as IQR/1.35. If data were not in a format directly amenable to meta-analysis, we adapted them according to the guidelines in Chapter 6 of the Cochrane Handbook for Systematic Reviews of Interventions (16). We conducted the analysis on an intention-to-treat basis for all included outcomes, analyzing all participants in the treatment groups to which they were randomized, irrespective of the actual treatment received.

Forest plots were constructed to visualize and assess treatment effects. Trial inconsistency was quantified using the I^2^ statistic, with significant inconsistency defined as I^2^ > 50% (17). Due to the limited number of studies identified (nine), we were unable to assess publication bias. Considering the limited amount of data in the article, we only performed subgroup analyses for the success rate at the first attempt, time required for successful intubation, and number of intubation attempts. Subgroup analyses were performed to examine potential differences in different clinical setting [operation room, Neonatal Intensive Care Unit (NICU), or other] and the expertise of the intubator (less experienced or intubation experts).

All analyses were performed with R software, version 4.0 (The R Foundation for Statistical Computing, https://www.r-project.org/). A two-sided *P* < 0.05 was considered statistically signiﬁcant.

### Trial sequential analysis

Trial Sequential Analysis (TSA), a robust statistical method tailored for repeated significance testing in cumulative meta-analyses, was employed to evaluate the primary and secondary outcome using TSA Module version 0.9.5.10 (Copenhagen Trial Unit, Copenhagen, Denmark). TSA is particularly valuable for addressing the risk of type I errors by determining the “information size”'—the necessary sample size for a meta-analysis beyond which adding additional studies is unlikely to alter the direction of the observed effect size. (18) For our analysis, we defined the alpha-spending boundary with an type I errors set at less than 5% and aimed for a statistical power of 80%. We applied both conventional boundaries (with an alpha of 5%) and adjusted O'Brien-Fleming boundaries (for random effects modeling with an alpha of 5% and a beta of 20%) for our outcomes of interest. The heterogeneity adjustment in the TSA was configured to a variance-based method under a random effects model. We constructed a cumulative, sequential *Z*-score curve by calculating the *Z*-statistic from each included trial. When the cumulative *Z*-score curve surpassed the TSA monitoring boundary, it would indicate that a conclusive result has been reached, allowing for confident decision-making based on the accumulated data.

## Results

### Study selection and characteristics

The initial comprehensive search identified 4,931 publications. After removing 1,262 duplicates, we screened 3,669 titles and abstracts, leading to the assessment of 61 full-text articles for eligibility. Ultimately, nine eligible RCTs were included in the meta-analysis (eFigure 1 in Supplementary 2) (8, 12, 19–25).

The characteristics of these trials are detailed in eTable 1. Collectively, the studies enrolled 1,059 patients, with 526 undergoing videolaryngoscopy and 533 receiving conventional direct laryngoscopy. The trials were published between 2009 and 2024 and included neonates of either sex undergoing endotracheal intubation at international centers located in Australia, Ireland, Indian, Canada, China, Egypt, the US, and the UK. Various videolaryngoscopes used in these studies included C-MAC, Airtraq, and Glidescope, with participants ranging from trainees to proficient providers such as neonatologists, pediatricians, or anesthesiologists. Only one study was a multicenter trial (19). For the intubation scenarios in the included studies, the settings varied across clinical environments: three studies were conducted in the NICU (8, 22, 24), four in the operating room (20, 21, 23, 25), one study was carried out either in the delivery room or NICU (12), and one took place in the neonatology ward (19).

### Risk of bias in included studies

**Figure 1:**
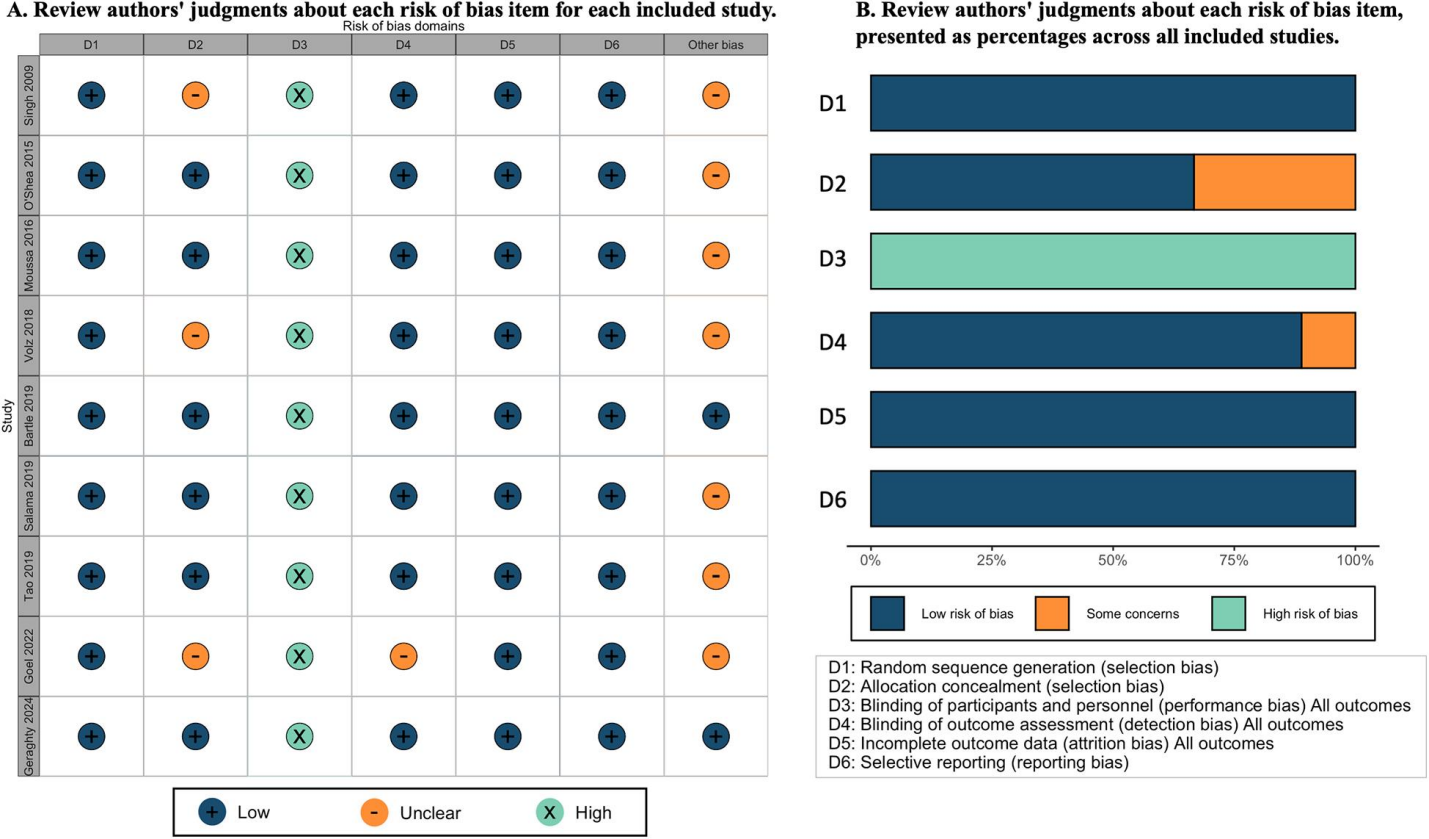
Risk of bias graph **(A)** and risk of bias summary **(B)**.

The risk of bias for the nine included studies is depicted in Figure 1. We assessed all nine studies as having a low risk of bias for randomization. Six studies were judged at low risk of bias (8, 12, 19–22), and three studies at an unclear risk for allocation concealment (23–25). Performance bias was considered high across all studies due to the inability to blind participants and providers to the intubation method used. In addition, in studies involving trainees, performance bias was exacerbated as participants were aware whether the supervisor could view the videolaryngoscope screen during the attempt (10, 22, 24) but unavoidable in such studies. One study was judged as unclear risk for outcome assessment due to no detail information was provide (25). All studies were deemed at low risk for attrition, and selective reporting bias. For a more detailed description of the risk of bias for each domain, refer to eTable 1 in Supplementary 2.

### Meta and TSA analysis

#### Primary outcome

**Figure 2:**
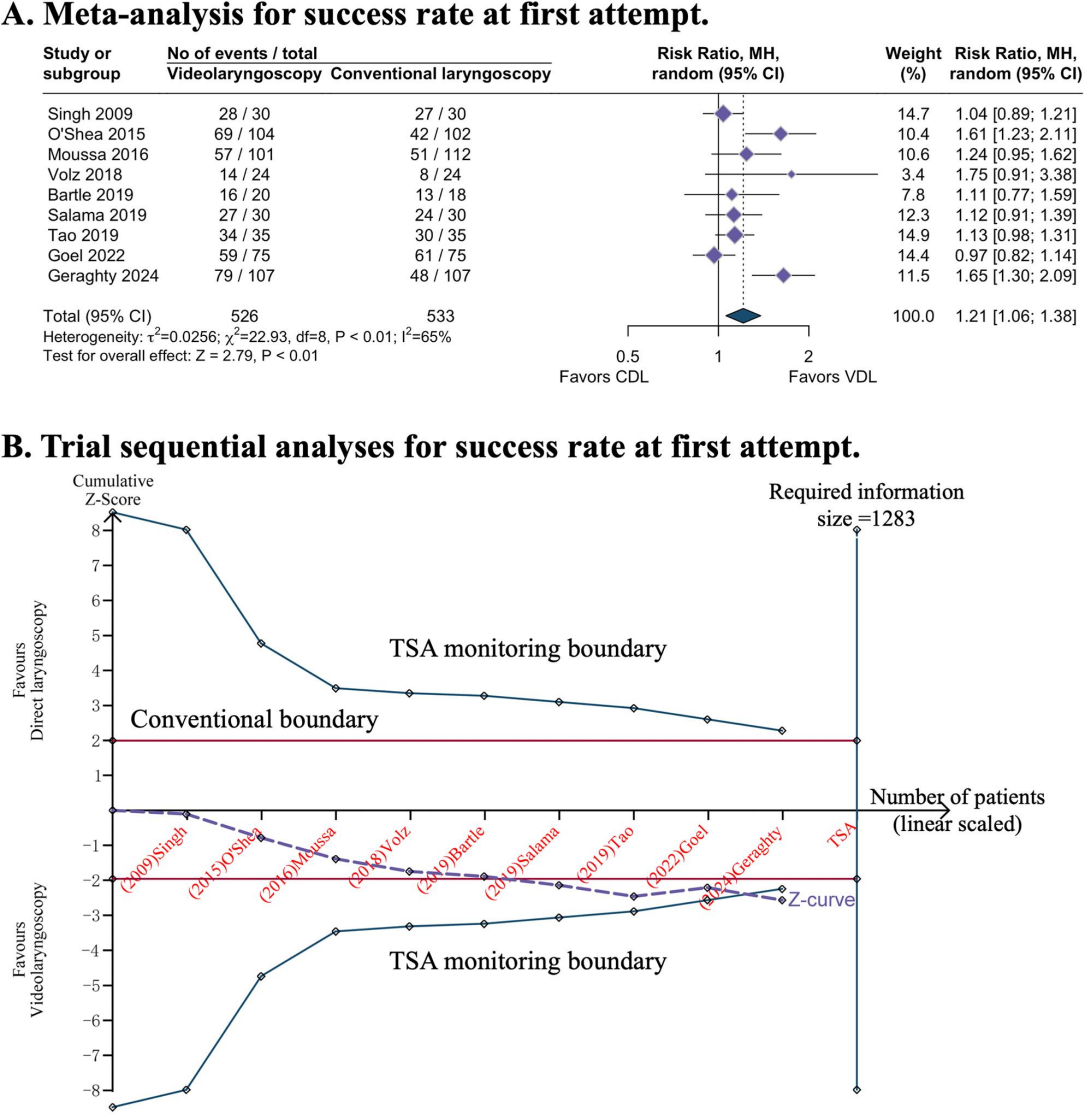
Success rate at first attempt with video or direct laryngoscopy. **(A)** Forest plot of meta-analysis. **(B)** Trial sequential analyses: error *α* = 5%, *β* = 20%, incidence in intervention arm = 71.8%, incidence in control arm = 53.1%%. CDL, conventional direct laryngoscopy; CI, confidence interval; TSA, trial sequential analyses; VDL, videolaryngoscopy.

All nine studies evaluated the primary outcome. Videolaryngoscopy enhanced the success of first attempt intubation [RR 1.21, 95% CI 1.06–1.38; 9 studies; I^2^ = 65%; 1,059 intubations (526 in videolaryngoscopy group, 533 in conventional direct laryngoscopy group); low-certainty evidence; Table 1 and Figure 2A]. TSA analysis indicated that the meta-analysis had significant evidence to support the outcome (Figure 2B, Table 1, and eTable 2).

**Table 1:** Videolaryngoscopy compared with conventional direct laryngoscopy for tracheal intubation in neonates.

Outcomes	Relative effect or Mean difference (95% CI)	No of intubations (studies)	*I* ^2^	Certainty of the evidence (GRADE)	TSA analysis				
					Cross TSMB	Cross FB	IS	Reach IS	Evidence
Success rate at first attempt	RR 1.21 (1.06–1.38)	1,059 (9)	65%	⊕⊕⊝⊝ Low^a^^,^^b^	Yes	No	1,283	No	Firm
Time required for successful intubation	MD 0.91 (−0.32–2.14)	805 (6)	97%	⊕⊕⊝⊝ Low^a^^,^^b^	Yes	No	625	Yes	Firm
Number of intubation attempts	MD −0.22 (−0.44–0.01)	959 (7)	65%	⊕⊕⊝⊝ Low^a^^,^^b^	Yes	No	723	Yes	Firm
Non-airway-related adverse effects: desaturation	RR 1.07 (0.89–1.30)	494 (4)	0%	⊕⊕⊕⊝ Moderate^b^	No	No	3,052	No	Absent
Non-airway-related adverse effects: bradycardia episodes	RR 1.15 (0.65–2.04)	647 (4)	8%	⊕⊝⊝⊝ Very low^a^^,^^b^^,^^c^	No	No	23,436	No	Absent
Non-airway-related adverse effects: lowest saturations during intubation	MD 0.05 (−0.12–0.21)	573 (3)	1%	⊕⊕⊕⊝ Moderate^b^	No	No	700	No	Absent
Airway-related adverse effects: airway trauma	RR 0.23 (0.06–0.89)	617 (5)	0%	⊕⊕⊕⊝ Moderate^b^	No	No	9,137	No	Absent
GRADE Working Group grades of evidence:									
High certainty: we are very confident that the true effect lies close to that of the estimate of the effect									
Moderate certainty: we are moderately confident in the effect estimate: the true effect is likely to be close to the estimate of the effect, but there is a possibility that it is substantially different									
Low certainty: our confidence in the effect estimate is limited: the true effect may be substantially different from the estimate of the effect									
Very low certainty: we have very little confidence in the effect estimate: the true effect is likely to be substantially different from the estimate of effect									

The basis for the assumed risk is the mean value across control groups. The corresponding risk (and its 95% confidence interval) is based on the assumed risk in the comparison group and the relative effect of the intervention (and its 95% CI), or the mean difference between the control and intervention groups, with its 95% CI.

CI, confidence interval; FB, futility boundary; MD, mean difference; RR: risk ratio; IS, information size; TSA, trial sequential analyses; TSMB, trial sequential monitoring boundary.

^a^ Downgraded one level for study limitations (due to high risk/unclear risk of bias).

^b^ Downgraded one level for serious inconsistency, such as high heterogeneity.

^c^ Downgraded one level for imprecision.

Subgroup analyses of three studies conducted in the NICU demonstrated that videolaryngoscopy significantly improved the success rate of first-attempt intubation (RR 1.44, 95% CI 1.16–1.80; eFigure 2A). This benefit was not observed in other clinical settings. Additionally, videolaryngoscopy appeared to have a higher success rate of first-attempt intubation (RR 1.51, 95% CI 1.28–1.78; eFigure 2B) when the intubations were conducted by less-experienced intubators.

#### Secondary outcomes

**Figure 3:**
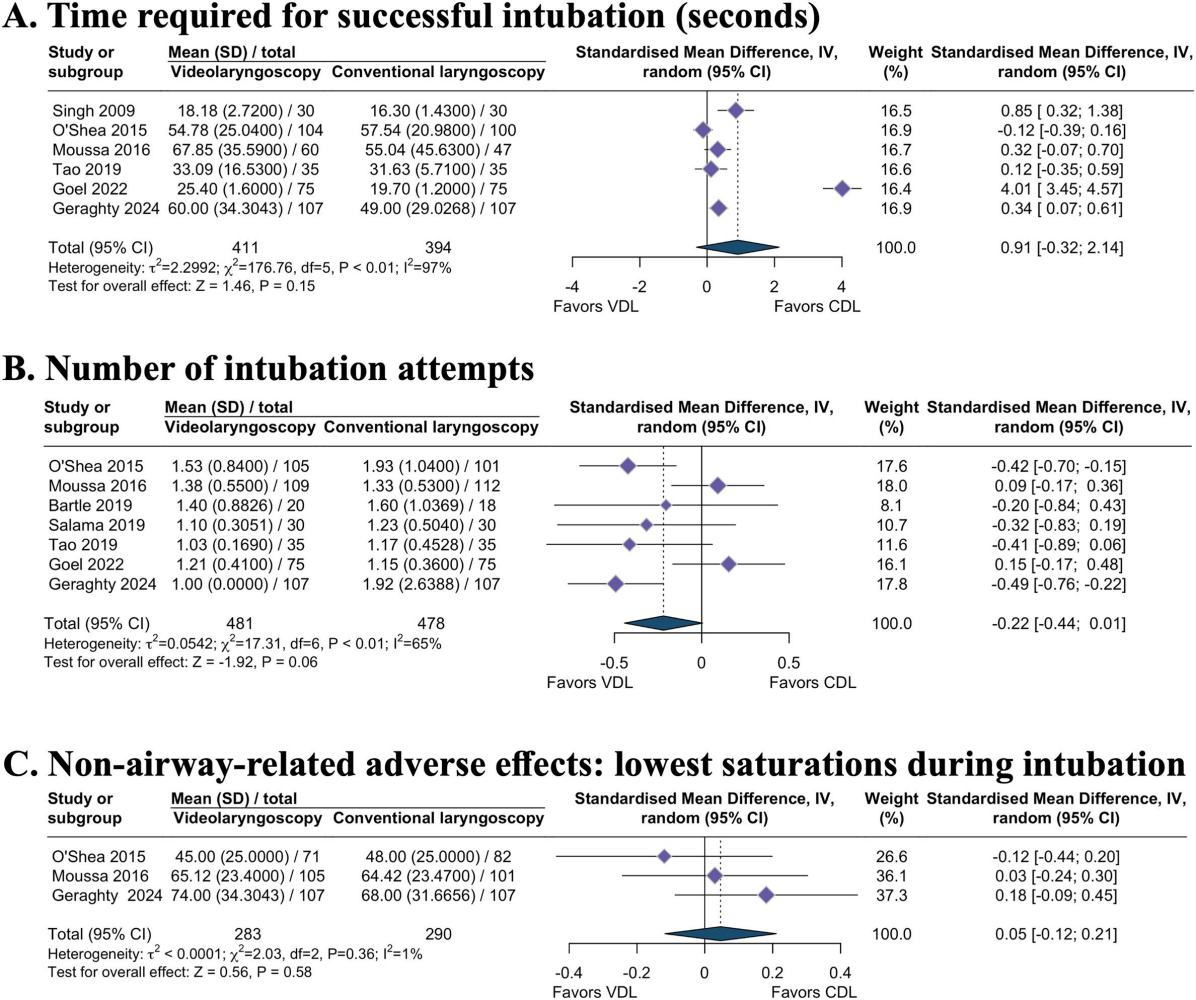
Secondary continuous outcomes. **(A)** Time required for successful intubation (seconds). **(B)** Number of intubation attempts. **(C)** Non-airway-related adverse effects: lowest oxygen saturation during intubation. VDL, videolaryngoscopy; CDL, conventional direct laryngoscopy; SMD, standardized mean difference; CI, confidence interval.

Six studies reported no significant reduction in time required for successful intubation when comparing videolaryngoscopy to direct laryngoscopy (MD 0.91, 95% CI −0.32–2.14; I^2^ = 97%; 6 studies; 805 intubations; low-certainty evidence; Table 1 and Figure 3A) (8, 12, 20, 22, 23, 25). TSA of pooled meta-analysis had ﬁrm evidence for anticipated intervention effect (Table 1 and eFigure 3). The intubation scenario (eFigure 4A) and the experience level of the intubator (eFigure 4B) did not influence the intubation time for the two groups.

Seven studies suggested that videolaryngoscopy did not reduce the number of intubation attempts (MD −0.22, 95% CI −0.44 to −0.01; I^2^ = 65%; 7 studies; 962 intubations; low-certainty evidence; Table 1 and Figure 3B) (8, 12, 19–22, 25). TSA indicated that this meta-analysis reached the requisite information size with firm evidence (Table 1 and eFigure 5). The intubation scenario (eFigure 6A) and the experience level of the intubator (eFigure 6B) did not influence the intubation time for the two groups.

**Figure 4:**
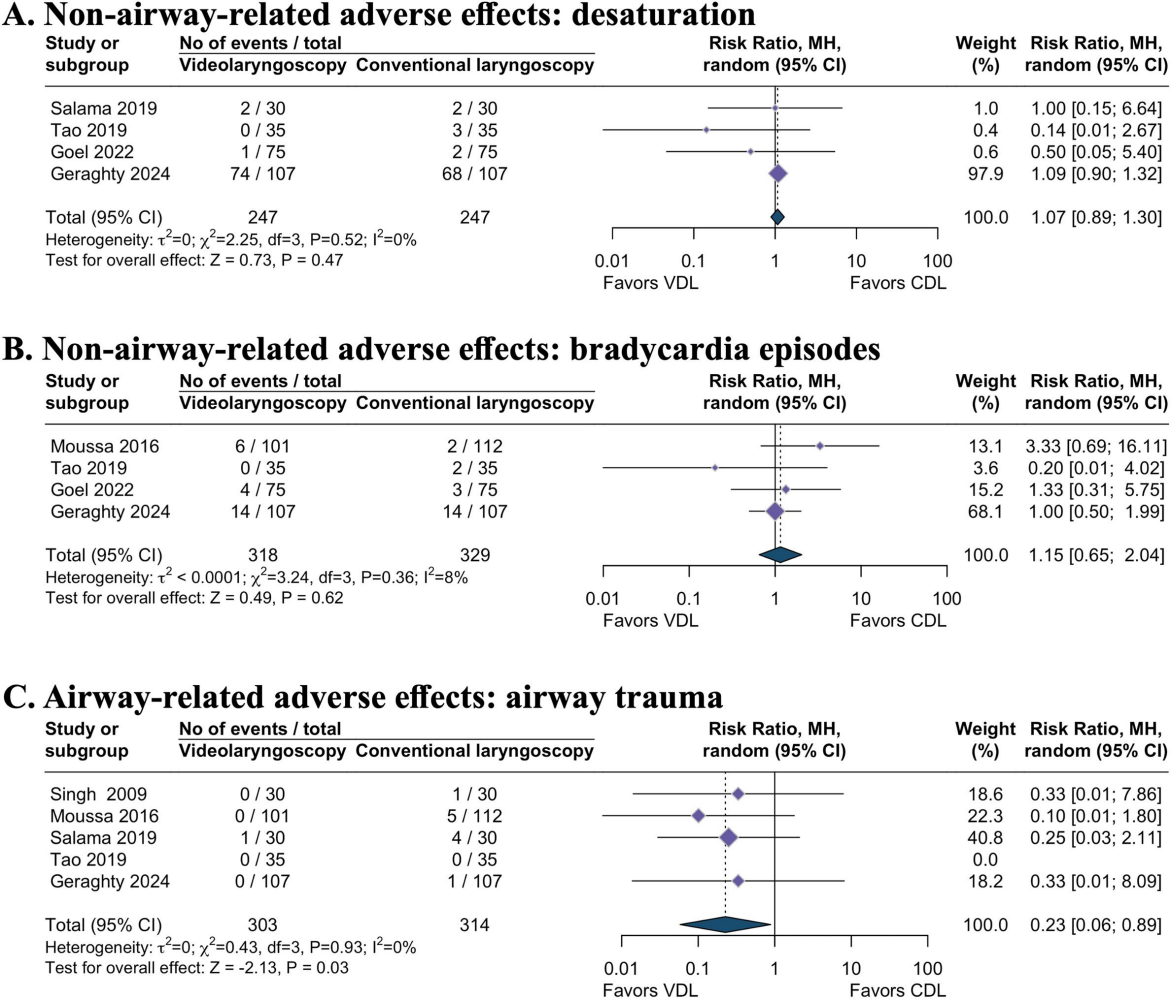
Secondary dichotomous outcomes. **(A)** Non-airway-related adverse effects: desaturation. **(B)** Non-airway-related adverse effects: bradycardia episodes. **(C)** Airway-related adverse effects: airway trauma. VDL, videolaryngoscopy; CDL, conventional direct laryngoscopy; RR, risk ratio; CI, confidence interval.

Evidence regarding the effect of videolaryngoscopy on desaturation (RR 1.07, 95% CI 0.89–1.30; 4 studies; I^2^ = 0%; 494 intubations; moderate-certainty evidence; Table 1 and Figure 4A) (12, 21, 22, 25), bradycardia episodes (RR 1.15, 95% CI 0.65–2.04; 4 studies; I^2^ = 8%; 647 intubations; very low-certainty evidence; Table 1 and Figure 4B) (8, 12, 20, 25), and lowest recorded O_2_ saturations during intubation (MD 0.05, 95% CI −0.12–0.21; I^2^ = 1%; 3 studies; 573 intubations; moderate-certainty evidence; Table 1 and Figure 3C) (8, 12, 22) remains uncertain. TSA analysis demonstrated that these outcomes have not yet reached the information size and lack firm evidence (Table 1).

Videolaryngoscopy likely resulted in a reduction of airway trauma incidence during intubation attempts compared to direct laryngoscopy (RR 0.23, 95% CI 0.06–0.89; 5 studies; I^2^ = 0%; 617 intubations; moderate-certainty evidence; Table 1 and Figure 4C) (8, 12, 20, 21, 23). However, TSA revealed the absence of conclusive evidence for the anticipated intervention effect (Table 1).

## Discussion

Our systematic review and meta-analysis incorporated nine RCTs that compared videolaryngoscopy with conventional, direct laryngoscopy for endotracheal intubation in neonates. The evidence indicated that videolaryngoscopy enhanced the success rate of first-attempt intubation, without significantly decreasing the number of intubation attempts or the time required for successful intubation. Additionally, videolaryngoscopy likely minimized the incidence of airway trauma. Our findings suggested that videolaryngoscopy was a preferable option for inexperienced clinicians performing neonatal intubation and for procedures conducted in the NICU. In terms of adverse events such as desaturation, bradycardic episodes, and the lowest recorded O_2_ saturations during intubation, videolaryngoscopy showed minimal difference compared to direct laryngoscopy. One limitation of our meta-analysis was the inability to analyze specific types of failed intubation events, such as esophageal intubation. Although esophageal intubation is a common adverse event during neonatal intubation procedures, none of the included RCTs specifically reported this outcome.

A concurrent systematic review, recently published by Lingappan and colleagues (11), also evaluated our primary question using meta-analysis and included eight studies, similar to ours. In our review, we restricted inclusion to trials published as full-text articles, excluding data from Kamath and colleagues as their study was only published as an abstract, making a full risk of bias assessment impossible due to insufficient details (26). While, we incorporated data from trial by Geraghty et al. that published in 2024 (12) and Goel et al. that published in 2022 (25). Despite these differences in inclusion criteria, both meta-analyses yielded some similar results. To enhance the robustness of our findings, we employed TSA to assess the adequacy of power and the risk of random error amid sparse data and potential updates. TSA confirmed solid evidence supporting the success rates of first-attempt intubation, the number of intubation attempts, and the duration required for successful intubation. It is crucial to distinguish between two different aspects of evidence assessment in our study. The TSA classification of “firm evidence” indicates that our cumulative sample size is sufficient to draw statistically reliable conclusions about the observed effect. However, this statistical adequacy should not be confused with the overall quality of evidence. Our GRADE ratings of “low” for several outcomes (first-attempt success, time, and number of attempts) reflect important methodological limitations in the contributing studies, particularly high performance bias. This means that while we have enough data to detect an effect, our confidence in the magnitude and reliability of this effect is limited by the quality of the underlying studies. This distinction is particularly relevant for clinical decision-making, as it suggests that while our findings are statistically robust, they should be interpreted with caution given the methodological limitations of the available evidence.

For first-attempt success, time to successful intubation, and number of intubation attempts, we observed substantial heterogeneity—likely driven by differing outcome definitions across studies (eTable 4)—which could not be fully resolved and therefore necessitated downgrading the GRADE certainty. Additional robust trials are still needed to consolidate these findings. Furthermore, TSA showed that the accrued information was insufficient to establish a definitive reduction in airway trauma with videolaryngoscopy, despite a moderate GRADE rating for this outcome. Notably, repeated endotracheal intubation attempts are associated with increased adverse events in neonates (2, 3). Our analysis showed a trend towards fewer intubations with videolaryngoscopy compared to conventional direct laryngoscopy, though not statistically significant. Additionally, our study lacked the power to detect significant effects on these adverse outcomes. Given the association between multiple intubation attempts and increased risk of complications, future studies should be conducted using a large sample size to robustly assess whether videolaryngoscopy can effectively reduce these risks. Such research is crucial for determining if the benefits of videolaryngoscopy extend beyond technical performance into clinically significant outcomes that will transform the standard care for neonatal intubation.

Previous studies indicated that for experienced intubators, success rates using videolaryngoscopy compared with direct laryngoscopy are as high or slightly higher in patients with normal airways (27, 28), and significantly higher in patients with anticipated difficult airways (28–30). Inexperienced intubators, when using videolaryngoscopes compared to direct laryngoscopes, also demonstrated greater success in intubating healthy adults with normal airways (31). But there is a lack of specific data on neonatal intubation related to this modifiable effect by intubation experience. Our meta-analysis observed that for clinicians with limited experience in intubation, the use of a videolaryngoscope significantly improves the success rate at the first attempt.

Current Neonatal Resuscitation Program (NRP) recommendations for neonate intubation only reference direct laryngoscopy and generally do not recommend or discuss videolaryngoscopy (32). Given the potential benefits highlighted by our findings, this practice should be reconsidered, especially considering the greater effectiveness and potentially reduced harm associated with videolaryngoscopy. More importantly, videolaryngoscopy was a better option for neonatal intubation by clinicians with limited intubatioin experience. Furthermore, the significantly higher cost of videolaryngoscopes compared to direct laryngoscopes poses substantial implications for their broader adoption. This economic factor must be carefully weighed in decisions about expanding the use of videolaryngoscopy in clinical practice.

We acknowledge several limitations in this systematic review and meta-analysis. The absence of definitive long-term data does not exclude a potential later difference between groups, representing a limitation of this analysis. Due to the limited number of studies included, we did not conduct sensitivity analysis according to the risk of bias or assess publication bias. We believe the following subgroups are justifiable due to the size of the airway and mouth (birth weight categories), the use of pre-medication vs. no pre-medication, and urgency (emergent or not), all of which may affect the success of the intubation procedure. While, currently, there is insufficient data to support these analyses. The incomplete reporting of laryngoscope brands and models across studies (see eTable 3) prevented us from conducting sensitivity analyses to evaluate the potential impact of hardware differences on outcomes. Future trials should consistently report detailed hardware specifications to enable assessment of device-specific effects. Finally, there is a lack of information on race or ethnicity in the included studies and that this is an important area for further study.

## Conclusion

The findings of this systematic review and meta-analysis suggested that videolaryngoscopy enhanced the success rate of first-attempt intubation and likely led to a decrease in the incidence of airway-related adverse effects, all while requiring a similar amount of time for successful intubation as direct laryngoscopy. These findings suggest that videolaryngoscopy could be more effective compared to direct laryngoscopy for endotracheal intubation in neonates.

## Data Availability

The original contributions presented in the study are included in the article/Supplementary Material, further inquiries can be directed to the corresponding authors.
