# Supplementary material for: Video vs. direct laryngoscopy for tracheal intubation in neonates: a meta-analysis
Source: Front Pediatr. 2025 Oct 23;13:1674255. doi: 10.3389/fped.2025.1674255 (PMC12589002; doi:10.3389/fped.2025.1674255)
Supplement: Supplementary file 1 [file Table1.docx]

**Search strategies**

**Search date:** June 2024

**MEDLINE OVID**

**Total: 689**

1 exp Intubation/

2 (laryngoscopy or laryngoscope).mp. [mp=title, abstract]

3 'direct laryngoscopy'.mp.

4 ('video laryngoscopy' or videolaryngoscopy).mp.

5 Glidescope.mp.

6 McGrath.mp.

7 (Pentax or 'airway scope').mp.

8 C-MAC.mp.

9 Truview.mp.

10 (Airtraq or 'optical layrngoscope').mp.

11 LMA CTrach.mp.

12 Neoview.mp. 0

13 1 or 2 or 3 or 4 or 5 or 6 or 7 or 8 or 9 or 10 or 11 or 12 65801

14 exp Infant, Premature, Diseases/ or exp Infant, Newborn/ or exp Infant/ or

'infant newborn'.mp.

15 neonat*.mp.

16 newborn*.mp.

17 14 or 15 or 16

18 13 and 17 9187

19 limit 18 to randomized controlled trial

**Embase OVID**

**Total: 772**

#1 ('intubation'/exp OR 'intubation' OR laryngoscopy OR laryngoscope OR 'direct laryngoscopy' OR 'video laryngoscopy' OR 'videolaryngoscopy' OR glidescope OR mcgrath:ta,ab,kw,ti OR pentax OR 'airway scope' OR 'c mac' OR truview OR airtraq OR 'optical laryngoscope' OR 'lma ctrach' OR neoview) AND ('infant - newborn'/exp OR 'infant - newborn' OR neonat OR newborn)

#2 #1 AND 'randomized controlled trial'/

**CINAHL Ebsco**

**Total: 1226**

(intubation or (laryngoscopy or laryngoscope) or 'direct laryngoscopy' or ('video laryngoscopy' or videolaryngoscopy)) and (infant or newborn or neonat* ) and ('randomized controlled trial' OR 'controlled clinical trial' OR randomized OR 'controlled clinical trial' or randomiz*'or randomis* or 'controlled clinical trials')

**Cochrane CENTRAL**

**Total: 2244**

#1 intubation

#2 laryngoscopy or laryngoscope

#3 'direct laryngoscopy'

#4 'video laryngoscopy' or videolaryngoscopy

#5 #1 or #2 or #3 or #4

#6 infant

#7 newborn

#8 neonat*

#9 #6 or #7 or #8

#10 #5 and #9
