# Supplementary material for: Video vs. direct laryngoscopy for tracheal intubation in neonates: a meta-analysis
Source: Front Pediatr. 2025 Oct 23;13:1674255. doi: 10.3389/fped.2025.1674255 (PMC12589002; doi:10.3389/fped.2025.1674255)
Supplement: Supplementary file 2 [file Datasheet1.docx]

**Supplemental Online Content**

**eTable 1. Characteristics of included studies.**

**eTable 2. Trial sequential analysis for all outcomes.**

**eTable 3. Different laryngoscope models**

**eTable 4. Definitions of First Attempt Success Rate, Intubation Time, and Number of Attempts**

**eFigure 1. Flow diagram of the identified trials.**

**eFigure 2. Subgroup analysis for the success rate at first attempt.**

**eFigure 3. Trial sequential analyses for time required for successful intubation.**

**eFigure 4. Subgroup analysis for** **time required for successful intubation.**

**eFigure 5. Trial sequential analyses for number of intubation attempts.**

**eFigure 6. Subgroup analysis for** **number of intubation attempts.**

**eTable 1. Characteristics of included studies**

| **Singh 2009** | | | | | | |
| --- | --- | --- | --- | --- | --- | --- |
| **Study characteristics** | | | | | | |
| **Region** | Indian | **Sample**  **size** | Videolaryngoscopy | 30 | **Research centers** | ☑ Single |
|  |  |  | Conventional laryngoscopy | 30 |  | ☐ Multicenter |
| **Participants** | | Neonates and infants of either sex undergoing surgery under general anesthesia, weighing 1 to 10kg. Exclusion criteria included raised intracranial pressure, high risk for pulmonary aspiration of gastric contents, such as gastric outlet obstruction and stasis, coagulopathy, and presence of any pathology of head and neck. 60 neonates and infants were enrolled in the study | | | | |
| **Interventions** | | Intubation performed using the Truview infant blade (videolaryngoscopy group; 30 neonates and infants) compared with the Miller blade number 0 (direct laryngoscopy group; 30 neonates and infants) | | | | |
| **Methods** | | Prospective randomized study to compare the Truview infant EVO2 laryngoscope with the Miller straight blade laryngoscope. The study was conducted in India. | | | | |
| **Outcomes** | | Number of attempts required for intubation, time for intubation, and the view of the glottis at laryngoscopy were scored according to the Cormack and Lehane grading criteria, were measured. Inability to intubate after three attempts was considered a failure to intubate. Hemoglobin, oxygen saturation, and all complications related to intubation were also recorded | | | | |
| **Notes** | | Funding sources/declarations of interest were not stated | | | | |
| **Risk of bias** | | | | | | |
| **Random sequence generation (selection bias)** | | Low risk (Randomization done by chit in a box technique) | | | | |
| **Allocation concealment (selection bias)** | | Unclear risk (No details given) | | | | |
| **Blinding of participants and personnel (performance bias) All outcomes** | | High risk (Participants could not be blinded) | | | | |
| **Blinding of outcome assessment (detection bias) All outcomes** | | Low risk (Time to intubation and intubation attempts were measured using objective Criteria) | | | | |
| **Incomplete outcome data (attrition bias) All outcomes** | | Low risk (All participants randomised to each group were included in the outcomes) | | | | |
| **Selective reporting (reporting bias)** | | Low risk (Investigators reported all outcomes that were specified in the protocol) | | | | |
| **Other bias** | | Unclear risk (Unknown) | | | | |
| **Salama 2019** | | | | | | |
| **Study characteristics** | | | | | | |
| **Region** | Egypt | **Sample**  **size** | Videolaryngoscopy | 32 | **Research centers** | ☑ Single |
|  |  |  | Conventional laryngoscopy | 32 |  | ☐ Multicenter |
| **Participants** | | American Society Anesthesiology physical status I or II neonates with normal craniofacial anatomy presenting for elective surgical repair of meningocele or myelomeningocele under general anesthesia. Exclusion criteria included the presence of suspected difficult intubation, high risk of pulmonary aspiration, and hemodynamic instability. | | | | |
| **Interventions** | | GlideScope videolaryngoscope compared to standard Miller direct laryngoscope. 60 neonates (30 in the videolaryngoscopy group and 30 in the direct laryngoscopy group) were included in the study. | | | | |
| **Methods** | | Prospective randomized study comparing videolaryngoscopy with GlideScope to direct laryngoscopy for tracheal intubation in laterally positioned neonates. Random allocation was performed using computer-generated numbers, using allocation concealment. The study was conducted in Egypt. | | | | |
| **Outcomes** | | The primary outcome was the laryngeal view, graded using the POGO score. Secondary outcomes were time to the best glottis view, endotracheal tube passage time, intubation time, POGO scores after optimal external laryngeal maneuvers, intubation attempts, and overall success rate of intubation. Any trauma caused during laryngoscopy and any decrease in oxygen saturation < 95% were recorded. | | | | |
| **Notes** | | There were no funding sources for the study and authors disclosed no conflicts of interest with the study. | | | | |
| **Risk of bias** | | | | | | |
| **Random sequence generation (selection bias)** | | Low risk (Randomization was performed by an independent statistician in a 1:1 ratio) | | | | |
| **Allocation concealment (selection bias)** | | Low risk (Allocation concealment was done by sequentially numbered, sealed, opaque envelopes.) | | | | |
| **Blinding of participants and personnel (performance bias) All outcomes** | | High risk (Participants could not be blinded.) | | | | |
| **Blinding of outcome assessment (detection bias) All outcomes** | | Low risk (Except for the percentage of glottic opening score, all data were recorded by  an independent observer not involved in the study.) | | | | |
| **Incomplete outcome data (attrition bias) All outcomes** | | Low risk (All participants randomised to each group were included in the outcomes.) | | | | |
| **Selective reporting (reporting bias)** | | Low risk (Investigators reported all outcomes that were specified in the protocol.) | | | | |
| **Other bias** | | Unclear (risk unknown) | | | | |
| **Moussa 2016** | | | | | | |
| **Study characteristics** | | | | | | |
| **Region** | Canada | **Sample**  **size** | Videolaryngoscopy | 18 | **Research centers** | ☑ Single |
|  |  |  | Conventional laryngoscopy | 19 |  | ☐ Multicenter |
| **Participants** | | All intubations attempted by residents in the NICU were included. Intubations performed on neonates with major oral, cervical, or upper airway malformations, emergency intubations, and unexpected difficult intubations (that needed an anesthetist to secure airway) were excluded from the study. Sample size based on power analysis was 100 intubations per group. 112 intubations were studied in the direct laryngoscopy group and 101 intubations in the video laryngoscopy group | | | | |
| **Interventions** | | Endotracheal intubation with conventional laryngoscope (Rusch, Teleflex Medical, Markham, Canada) with Miller blade size 00, 0, or 1, or the videolaryngoscope C-MAC VL (Karl Storz, Tuttlingen, Germany) with blade size 0 or 1 | | | | |
| **Methods** | | A non-blinded, randomized controlled trial with cross-over of only the experimental group in the second phase. All intubations were performed with direct laryngoscopy in the second phase. The study was conducted in Canada. Randomization was by concealed allocation using a table of random numbers stratified by year of residency training | | | | |
| **Outcomes** | | Primary outcome: success rate of endotracheal intubation, defined as correct anatomical placement of the ETT in 3 attempts (confirmed by change in color of the carbon dioxide detector, vapor in the endotracheal tube, thoracic expansion, assessment of bilateral lung air entry, absence of air entry in the stomach by auscultation, and improvement of neonate's clinical parameters).Secondary outcomes: number of attempts, time to successful intubation, number of bradycardia episodes and lowest oxygen saturation during procedure, mucosal trauma, number of failures, and reason for intubation failure | | | | |
| **Notes** | | Performed at the University of Montreal, Canada. Study was funded by American Academy of Pediatrics Neonatal Resuscitation Program Young Investigator Award and a Medical Education Research Grant from Direction de l'enseignement, Centre Hospotalier Universitaire Sainte-Justine.Nondedicated educational grant from Ikaria Canada Inc. used to purchase the videolaryngoscope.Karl-Storz Endoscopy loaned equipment (videolaryngoscope intubation blades) for this study, although they were not involved in study design, data collection, analysis, or publication. Ikaria Canada Inc. was not involved in study design, data collection, analysis, or publication. The authors declared no potential conflicts of interest. | | | | |
| **Risk of bias** | | | | | | |
| **Random sequence generation (selection bias)** | | Low risk (Table of random numbers stratified by year of residency training) | | | | |
| **Allocation concealment (selection bias)** | | Low risk (Sealed envelopes with the allocation group) | | | | |
| **Blinding of participants and personnel (performance bias) All outcomes** | | High risk (Study could not be blinded) | | | | |
| **Blinding of outcome assessment (detection bias) All outcomes** | | Low risk (Change in color of the carbon dioxide detector, vapor mist in the endotracheal tube, thoracic expansion, assessment of bilateral lung air entry, absence of air entry in the stomach by auscultation, and improvement of clinical parameters were used as objective criteria to assess intubation success) | | | | |
| **Incomplete outcome data (attrition bias) All outcomes** | | Low risk (34 out of 37 enrolled residents completed the study; outcomes were reported for 34 residents) | | | | |
| **Selective reporting (reporting bias)** | | Low risk (Investigators reported all outcomes that were specified in the protocol) | | | | |
| **Other bias** | | Unclear risk (More than two-thirds of intubations were nasotracheal) | | | | |
| **O'Shea 2015** | | | | | | |
| **Study characteristics** | | | | | | |
| **Region** | Australia | **Sample**  **size** | Videolaryngoscopy | 107 | **Research centers** | ☑ Single |
|  |  |  | Conventional laryngoscopy | 106 |  | ☐ Multicenter |
| **Participants** | | Infants without facial or airway anomalies in the delivery room or intensive care unit. Infants were eligible if they needed intubation, and the intervention was going to be performed orally by a pediatric resident in their first 6 months of tertiary neonatal training. The unit of randomization was the intubation attempt, and only the first attempt was analyzed. 206 intubations were assessed. | | | | |
| **Interventions** | | All intubations were performed using the modified traditional Miller videolaryngoscope (LaryFlex, Acutronics, Hirzel, Switzerland) with the videolaryngoscope screen either visible to the instructor or covered during intubation. 104 intubations were assessed in the group with the video laryngoscopy screen visible to the instructor, and 102 intubations were performed with the videolaryngoscopy screen covered | | | | |
| **Methods** | | Randomized controlled trial to compare intubation success by physicians with < 6 months' tertiary neonatal experience in neonates, with or without the videolaryngoscope screen visible to the instructor. Randomization was achieved through computer-generated, variable-size block-randomization sequence, and allocation concealment was used. The study was conducted in Australia | | | | |
| **Outcomes** | | First-attempt intubation success rate confirmed by colorimetric detection of expired carbon dioxide. Secondary outcomes included the infant’s lowest heart rate, oxygen saturation, and duration of the attempt. | | | | |
| **Notes** | | Single-center, unblinded study conducted in 2013 to 2014 at the Royal Women's Hospital, Melbourne, Australia, a tertiary perinatal centre. Funded by The Royal Women’s Hospital, Melbourne, Australia, and the Australian National Health and Medical Research Council Program (grant 606789). In Vitro Technologies provided a videolaryngoscope for the duration of the study but had no part in study design or data analysis. The authors indicated they had no financial relationships or conflicts of interest relevant to this article to disclose. | | | | |
| **Risk of bias** | | | | | | |
| **Random sequence generation (selection bias)** | | Low risk (Computer-generated, variable-sized, block randomization sequence used) | | | | |
| **Allocation concealment (selection bias)** | | Low risk (Sequentially numbered, opaque envelopes with randomization cards) | | | | |
| **Blinding of participants and personnel (performance bias) All outcomes** | | High risk (Participant could potentially know if the supervisor had access to the videolaryngoscope screen during their attempt, which could impact their performance) | | | | |
| **Blinding of outcome assessment (detection bias) All outcomes** | | Low risk (Primary outcome was success at the intubation attempt. Endotracheal tube placement was confirmed by a colorimetric exhaled carbon dioxide detector,  which is an objective criterion to assess intubation success) | | | | |
| **Incomplete outcome data (attrition bias) All outcomes** | | Low risk (After randomization, only 7 neonates were excluded (3 in the intervention group and 4 in the control group); analysis on an intention-to-treat basis) | | | | |
| **Selective reporting (reporting bias)** | | Low risk (Investigators reported all outcomes that were specified in the protocol) | | | | |
| **Other bias** | | Unclear risk (unknown) | | | | |
| **Bartle 2019** | | | | | | |
| **Study characteristics** | | | | | | |
| **Region** | England | **Sample**  **size** | Videolaryngoscopy | 21 | **Research centers** | ☐ Single |
|  |  |  | Conventional laryngoscopy | 18 |  | ☑ Multicenter |
| **Participants** | | Any infant (either sex) who required oral intubation was eligible for inclusion. Infants were not recruited if they were in extremis requiring immediate intubation by a senior experienced operator who used his/her own preferred method. Any baby with a congenital airway malformation was not included. Some babies were excluded as they were felt to be too small for the equipment to be effective. None of the procedures were carried out in emergency delivery circumstances. The sample size was 40 intubations. | | | | |
| **Interventions** | | Videolaryngoscopy with Storz C-MAC videolaryngoscope (size 1 or size 0 Miller blade) compared to direct laryngoscopy with standard neonatal laryngoscope. 40 intubations were performed in 39 babies (21 babies in the video laryngoscopy group and 18 in the direct laryngoscopy group). | | | | |
| **Methods** | | Feasibility study in which neonates were randomised to videolaryngoscopy with C-MAC or direct laryngoscopy for intubation. Randomized controlled trial, with randomization performed using concealment allocation. The study was performed at two centers in the United Kingdom. | | | | |
| **Outcomes** | | Number of attempts to successful intubation (initially determined clinically and then confirmed by chest radiograph with or without capnography) and confidence of accurate tube placement (trainee, trainer, and supporting staff confidence). Author was contacted and provided number of intubation attempts for each group. | | | | |
| **Notes** | | Performed across two sites: the local neonatal unit at Royal Devon and Exeter NHS Foundation Trust; the neonatal intensive care unit at Derriford Hospital, Plymouth. Funding sources/declarations of interest were not stated | | | | |
| **Risk of bias** | | | | | | |
| **Random sequence generation (selection bias)** | | Low risk (Statistician provided a block randomization list, which ensured that the envelopes were distributed evenly between the two units.) | | | | |
| **Allocation concealment (selection bias)** | | Low risk (Randomization was performed using concealment of allocation) | | | | |
| **Blinding of participants and personnel (performance bias) All outcomes** | | High risk (Participants could not be blinded.) | | | | |
| **Blinding of outcome assessment (detection bias) All outcomes** | | Low risk (Successful intubation was determined clinically, and subsequently confirmed by a chest radiograph with or without capnography. The research and development team performed independent data collection and entry of the data.) | | | | |
| **Incomplete outcome data (attrition bias) All outcomes** | | Low risk (One participant who was allocated to the control group was withdrawn. Outcomes were published for the remaining participants.) | | | | |
| **Selective reporting (reporting bias)** | | Low risk (Investigators reported all outcomes that were specified in the protocol. One participant in the experimental group was not included in the author's unpublished report of the number of intubation attempts.) | | | | |
| **Other bias** | | Low risk (Not at risk) | | | | |
| **Tao 2019** | | | | | | |
| **Study characteristics** | | | | | | |
| **Region** | China | **Sample**  **size** | Videolaryngoscopy | 35 | **Research centers** | ☑ Single |
|  |  |  | Conventional laryngoscopy | 35 |  | ☐ Multicenter |
| **Participants** | | American Society of Anesthesiologists physical status I and II neonates scheduled to undergo elective surgery under general anesthesia. Neonates with increased intracranial pressure or increased risk of aspiration were excluded. 70 neonates of either sex was enrolled in the study. | | | | |
| **Interventions** | | GlideScope videolaryngoscope using size 1 blade compared to direct laryngoscope_using_Macintosh size 1 blade. 70 neonates were enrolled in the study (35 in the videolaryngoscopy group and 35 in the direct laryngoscopy group). | | | | |
| **Methods** | | Randomized study comparing videolaryngoscopy using GlideScope to direct laryngoscopy. Stratified block randomization and allocation concealment were used. This study was conducted in China. | | | | |
| **Outcomes** | | The primary outcome was time to intubation. Secondary outcomes were the success rate of the first intubation attempt, number of intubation attempts, Cormack and Lehane grade of glottis view, and adverse events (trauma, desaturation, or bradycardia episodes). | | | | |
| **Notes** | | The study had no sources of funding support, and authors declared no conflicts of interest. | | | | |
| **Risk of bias** | | | | | | |
| **Random sequence generation (selection bias)** | | Low risk (Stratified blocked randomization) | | | | |
| **Allocation concealment (selection bias)** | | Low risk (An envelope containing the allocation information was given to the anesthesiologist  who performed the intubation, and the anesthesiologist opened the  envelope immediately before induction of anesthesia.) | | | | |
| **Blinding of participants and personnel (performance bias) All outcomes** | | High risk (Participants could not be blinded.) | | | | |
| **Blinding of outcome assessment (detection bias) All outcomes** | | Low risk (An observer, who was blinded to neonate group assignment, was responsible  for recording the time to intubation.) | | | | |
| **Incomplete outcome data (attrition bias) All outcomes** | | Low risk (All participants randomized to each group were included in the outcomes.) | | | | |
| **Selective reporting (reporting bias)** | | Low risk (Investigators reported all outcomes that were specified in the protocol.) | | | | |
| **Other bias** | | Unclear risk (unknown) | | | | |
| **Volz 2018** | | | | | | |
| **Study characteristics** | | | | | | |
| **Region** | United States | **Sample**  **size** | Videolaryngoscopy | 24 | **Research centers** | ☑ Single |
|  |  |  | Conventional laryngoscopy | 24 |  | ☐ Multicenter |
| **Participants** | | First- and second-year pediatric residents were approached for inclusion in the study. Exclusion criteria included residents who declined participation in the study. 48 residents (with 24 in each group) were randomized. | | | | |
| **Interventions** | | Study intervention was resident coaching during intubation, with or without the use of video as a coaching tool; residents were identified as the study subjects. _Videolaryngoscopy was performed using the C-MAC laryngoscope with Miller blade size 0 or 1 (Karl Storz Co.), and direct laryngoscopy was performed using the Rusch laryngoscope with Miller blade size 00, 0, or 1 (Teleflex, Morrisville, NC). 101 non-emergent intubations were analyzed (61 in the videolaryngoscopy group and 40 in the direct laryngoscopy group). | | | | |
| **Methods** | | Randomized controlled trial to compare intubation success by first and second-year pediatric residents in neonates, with or without guidance from the supervisor using videolaryngoscopy. 1:1 randomization and allocation concealment were used. This study was conducted in the United States. | | | | |
| **Outcomes** | | Successful endotracheal intubation (defined as the placement of an endotracheal tube in the infant’s trachea) within two attempts. Intubations were confirmed by auscultation of breath sounds, observation of equal chest wall movement, change in a colorimetric carbon dioxide detector, presence of mist in the endotracheal tube, and chest radiograph. | | | | |
| **Notes** | | There was no funding support for the study, and the authors disclosed no conflicts of interest. | | | | |
| **Risk of bias** | | | | | | |
| **Random sequence generation (selection bias)** | | Low risk (Residents were randomized by pulling concealed names out of a box using a 1:1 randomization scheme.) | | | | |
| **Allocation concealment (selection bias)** | | Unclear risk (No details given) | | | | |
| **Blinding of participants and personnel (performance bias) All outcomes** | | High risk (Participants were not blinded to their assigned groups.) | | | | |
| **Blinding of outcome assessment (detection bias) All outcomes** | | Low risk (Investigators who performed data analysis were blinded to the participants’ assigned study groups.) | | | | |
| **Incomplete outcome data (attrition bias) All outcomes** | | Low risk (No participants were excluded) | | | | |
| **Selective reporting (reporting bias)** | | Low risk (Investigators reported all outcomes that were specified in the protocol.) | | | | |
| **Other bias** | | Unclear (risk unknown) | | | | |
| **Goel 2022** | | | | | | |
| **Study characteristics** | | | | | | |
| **Region** | India | **Sample**  **size** | Videolaryngoscopy | 75 | **Research centers** | ☑ Single |
|  |  |  | Conventional laryngoscopy | 75 |  | ☐ Multicenter |
| **Participants** | | Inclusion criteria were all neonates of either gender of age 0‑28 days, American Society of Anaesthesiologists physical status (ASA‑PS) I and II and birth weight more than 1.5 kg. All neonates with anticipated difficult airway or with any congenital defects of the upper airway, head, and neck surgery, coagulation defects, birth asphyxia, prematurity.and risk of pulmonary aspiration were excluded from the study | | | | |
| **Interventions** | | One group undergoing intubation with either Miller blade, C‑MAC VL size 0 or1 (n = 75) and the intubation in the other group with the conventional Miller blade size 0 or 1 (n = 75). | | | | |
| **Methods** | | In the operating room, neonates were randomized into two groups using computer‑generated randomization labels. The single-center, randomized clinical trial was conducted in India. | | | | |
| **Outcomes** | | The outcomes include the time to best glottic view (measured as the time taken from the touching of the tip of the laryngoscope to the lip of the neonate till the time to best glottic view was achieved) and the time to intubation (assessed from the time the laryngoscope entered the neonate’s mouth until the first capnograph trace was seen on the monitor.) and the number of intubation attempts | | | | |
| **Notes** | | Authors disclosed no conflicts of interest with the study | | | | |
| **Risk of bias** | | | | | | |
| **Random sequence generation (selection bias)** | | Low (All neonates eligible for inclusion were included in the trial) | | | | |
| **Allocation concealment (selection bias)** | | Unclear risk (The article does not mention the relevant details) | | | | |
| **Blinding of participants and personnel (performance bias) All outcomes** | | High risk (Participants could not be blinded.) | | | | |
| **Blinding of outcome assessment (detection bias) All outcomes** | | Unclear risk (The article does not mention the relevant details) | | | | |
| **Incomplete outcome data (attrition bias) All outcomes** | | Low (Evaluation of the trial outcomes included all participants) | | | | |
| **Selective reporting (reporting bias)** | | Low (Investigators reported all outcomes) | | | | |
| **Other bias** | | Unclear risk (The article does not mention the relevant details) | | | | |
| Geraghty 2024 | | | | | | |
| **Study characteristics** | | | | | | |
| **Region** | Ireland | **Sample**  **size** | Videolaryngoscopy | 107 | **Research centers** | ☑ Single |
|  |  |  | Conventional laryngoscopy | 107 |  | ☐ Multicenter |
| **Participants** | | Neonates of any gestational age in whom intubation was attempted in the delivery room (i.e., labor or operating room) or neonatal intensive care unit were eligible for inclusion. Neonates who had upper airway anomalies were ineligible. Intubated neonates who were transferred from other hospitals were eligible for inclusion if they were subsequently intubated at the National Maternity Hospital. | | | | |
| **Interventions** | | In intubations that were performed in the video-laryngoscopy group, a video laryngoscope and Miller laryngoscope blades were used to visualize the airway. In the direct-laryngoscopy group, a standard laryngoscope(HEINE Optotechnik) and straight laryngoscope blades were used to visualize the airway directly through the mouth. | | | | |
| **Methods** | | Randomization was stratified according to gestational age at birth. The group assignment schedule was generated in blocks of four with the use of a random numbers table. The group assignments were written on cards and placed in sealed, opaque, sequentially numbered envelopes in a box that was kept in the neonatal intensive care unit. The single-center, randomized clinical trial was conducted in Dublin. | | | | |
| **Outcomes** | | The primary outcome was successful intubation on the first attempt. Secondary outcomes were the lowest oxygen saturation, the lowest heart rate during the intubation attempt, the number of attempts made to intubate successfully, the du-ration of successful attempts, and the correct positioning of the endotracheal tubes on a chest radiograph. | | | | |
| **Notes** | | Funding sources/declarations of interest were not stated | | | | |
| **Risk of bias** | | | | | | |
| **Random sequence generation (selection bias)** | | Low risk (Randomization was stratified) | | | | |
| **Allocation concealment (selection bias)** | | Low risk (The group assignments were written on cards and placed in sealed, opaque, sequentially numbered envelopes in a box that was kept in the neonatal intensive care unit) | | | | |
| **Blinding of participants and personnel (performance bias) All outcomes** | | High risk (Participants could not be blinded.) | | | | |
| **Blinding of outcome assessment (detection bias) All outcomes** | | Low risk (The outcomes are evaluated by objective measuring tool such as colorimetric exhaled carbon dioxide detector) | | | | |
| **Incomplete outcome data (attrition bias) All outcomes** | | Low risk (All patients were included in the analysis of outcomes) | | | | |
| **Selective reporting (reporting bias)** | | Low risk (Investigators reported all outcomes that were specified in the protocol.) | | | | |
| **Other bias** | | Low risk (The National Maternity Hospital Foundation (which funded the trial) and the manufacturer of the video laryngoscope had no role in designing or conducting the trial; collecting, analyzing, or interpreting the data; or making the decision to present or publish the results) | | | | |

**eTable 2. Trial sequential analysis for all outcomes.**

| **Parameters** |  |  | **Dichotomous outcomes** | | **Continuous outcomes** | |  |
| --- | --- | --- | --- | --- | --- | --- | --- |
|  | **α%** | **1-β%** | **Incidence in intervention arm, %** | **Incidence in control arm, %** | **Mean difference in intervention arm** | **Mean difference in control arm** | **Diversity, %** |
| **Success rate at first attempt** | 5 | 80 | 71.8 | 53.1 |  |  | 77.63 |
| **Desaturation** | 5 | 80 | 25.8 | 30.4 |  |  | 0 |
| **Bradycardia episodes** | 5 | 80 | 7.5 | 6.4 |  |  | 19.04 |
| **Airway trauma** | 5 | 80 | 2.5 | 3.5 |  |  | 0 |
| **Time required for successful intubation** | 5 | 80 | - | - | 43.22 seconds | 38.20 seconds | - |
| **Number of intubation attempts** | 5 | 80 | - | - | 1.24 | 1.48 | - |
| **Lowest oxygen saturation during intubation** | 5 | 80 | - | - | 61.37% | 60.04% | - |

**eTable 3. Different laryngoscope models**

| **Author** | **Tao** | **Salama** | **Goel** | **Singh** | **O’Shea** | **Moussa** | **Volz** | **Geraghty** | **Bartle** |
| --- | --- | --- | --- | --- | --- | --- | --- | --- | --- |
| **Year** | 2018 | 2019 | 2022 | 2009 | 2015 | 2016 | 2019 | 2024 | 2019 |
| **Video** | GlideScope Cobal | GlideScope Cobalt | C-MAC | TruView infant EVO2 | LaryFlex | C-MAC | C-MAC | C-MAC | C-MAC |
| **Direct** | Macintosh | NA | NA | NA | NA | Rusch | Rusch | HEINE Optotechnik | NA |

NA: Not Avaliable

**eTable 4. Definitions of First Attempt Success Rate, Intubation Time, and Number of Attempts**

| **Author** | **Year** | **Success rate at first attempt** | **Time required for successful intubation** | **Number of intubation attempts** |
| --- | --- | --- | --- | --- |
| Moussa | 2016 | Successful placement of ETT into trachea within ≤3 attempts | From insertion of laryngoscope blade into the mouth until its removal | Each insertion of laryngoscope blade into the mouth counted as one attempt |
| O’Shea | 2015 | Successful intubation on the first attempt confirmed by CO₂ detection | From insertion of laryngoscope blade into the mouth until its removal | One attempt defined as blade insertion; attempt terminated if >60s or desaturation/bradycardia occurred |
| Salama | 2019 | Successful intubation at the first attempt | From laryngoscope insertion into the mouth until its removal after tube placement; also recorded TBGV and TPT | Withdrawing tube to the angle of mouth and reintroducing counted as new attempt; failure if >3 attempts or >60s |
| Singh | 2009 | Successful intubation on the first attempt | From insertion of instrument into the mouth until its removal after ETT placement | Each laryngoscope insertion counted as one attempt; failure defined as unsuccessful after 3 attempts |
| Tao | 2018 | Successful intubation on the first attempt confirmed by EtCO₂ detection | From placement of blade between lips until EtCO₂ detection | Each blade insertion into lips counted as one attempt; if failed after 2 attempts, other methods used |
| Volz | 2019 | Successful intubation on the first attempt | Length of attempt: from laryngoscope insertion into the mouth until its removal | Each laryngoscope insertion counted as one attempt; successful if ETT placed within ≤2 attempts |

**
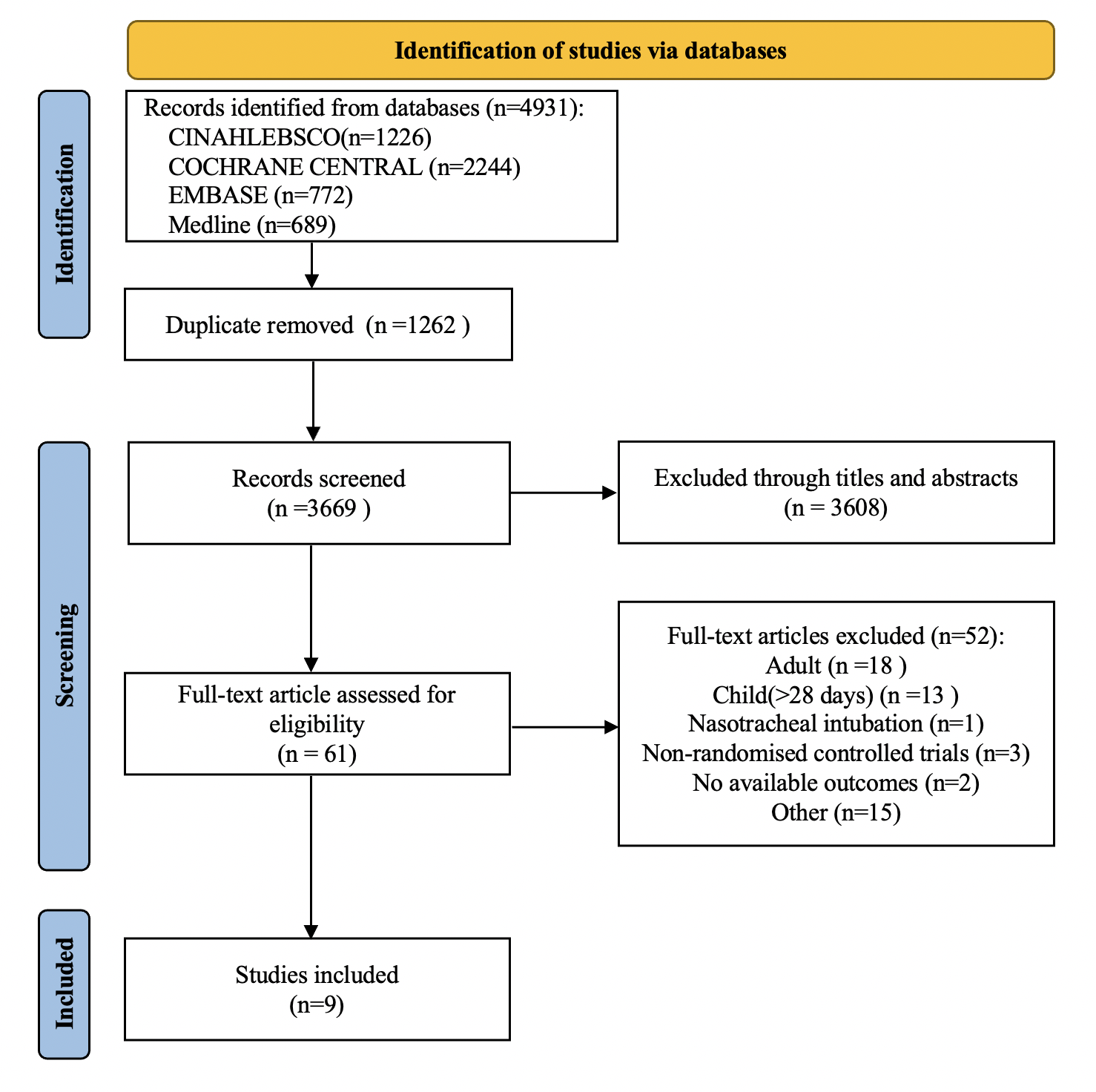
eFigure 1. Flow diagram of the identified trials.**


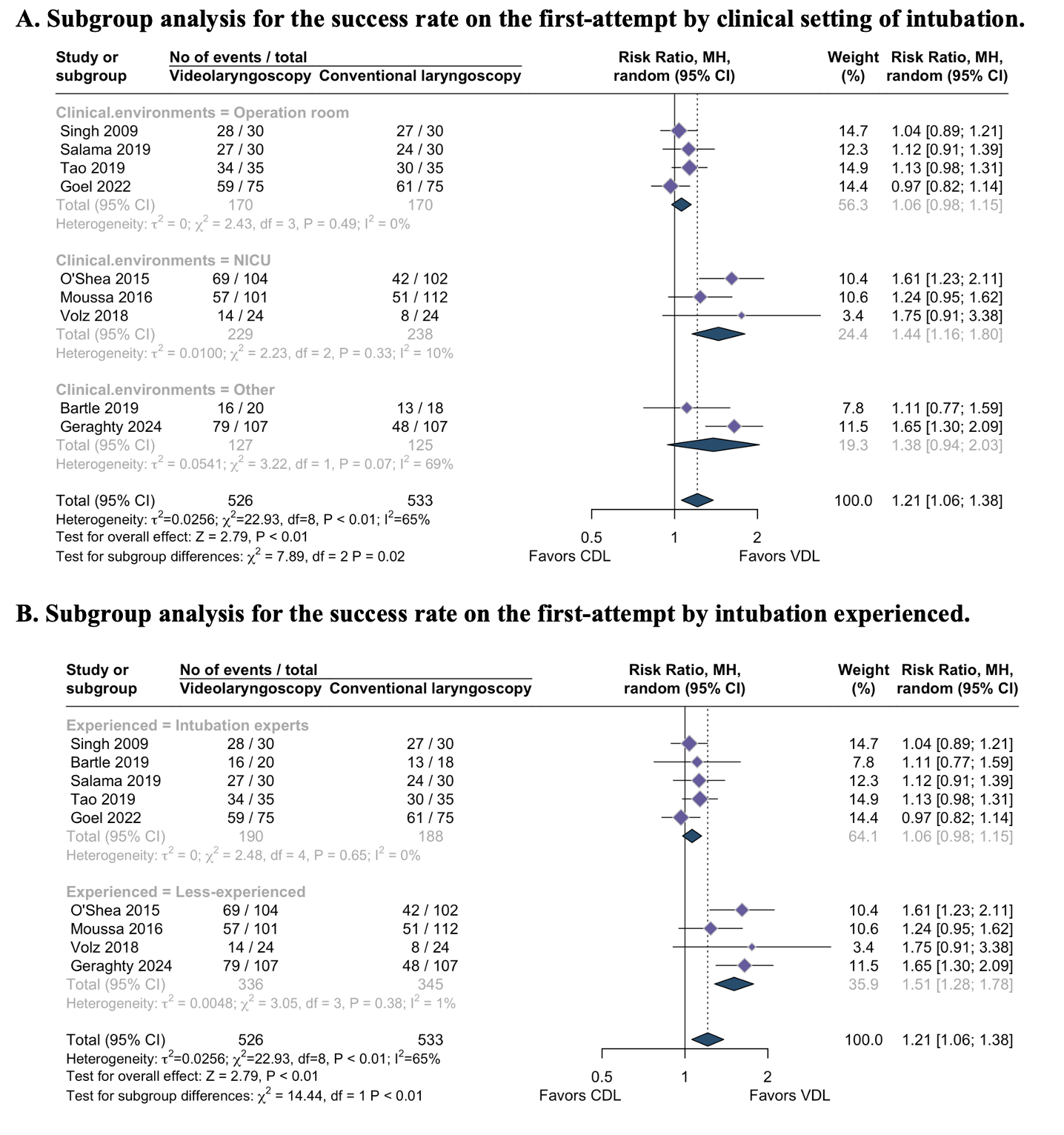
**eFigure 2. Subgroup analysis for the success rate at first attempt.**

Abbreviation: CDL, conventional direct laryngoscopy; CI, confidence interval; NICU, Neonatal Intensive Care Unit; VDL, Videolaryngoscopy.


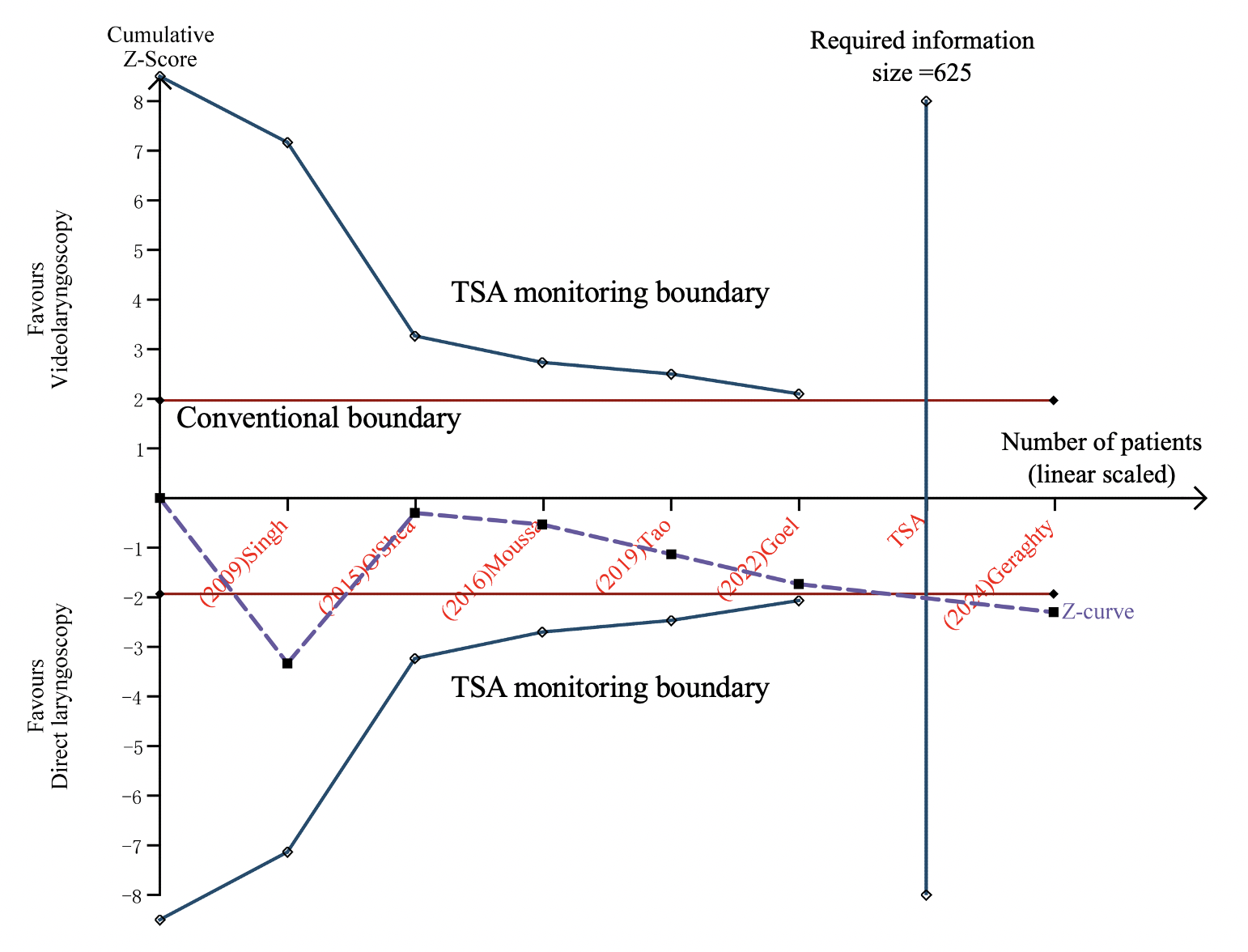
**eFigure 3. Trial sequential analyses for time required for successful intubation.**

Abbreviation: TSA, Trial sequential analyses.


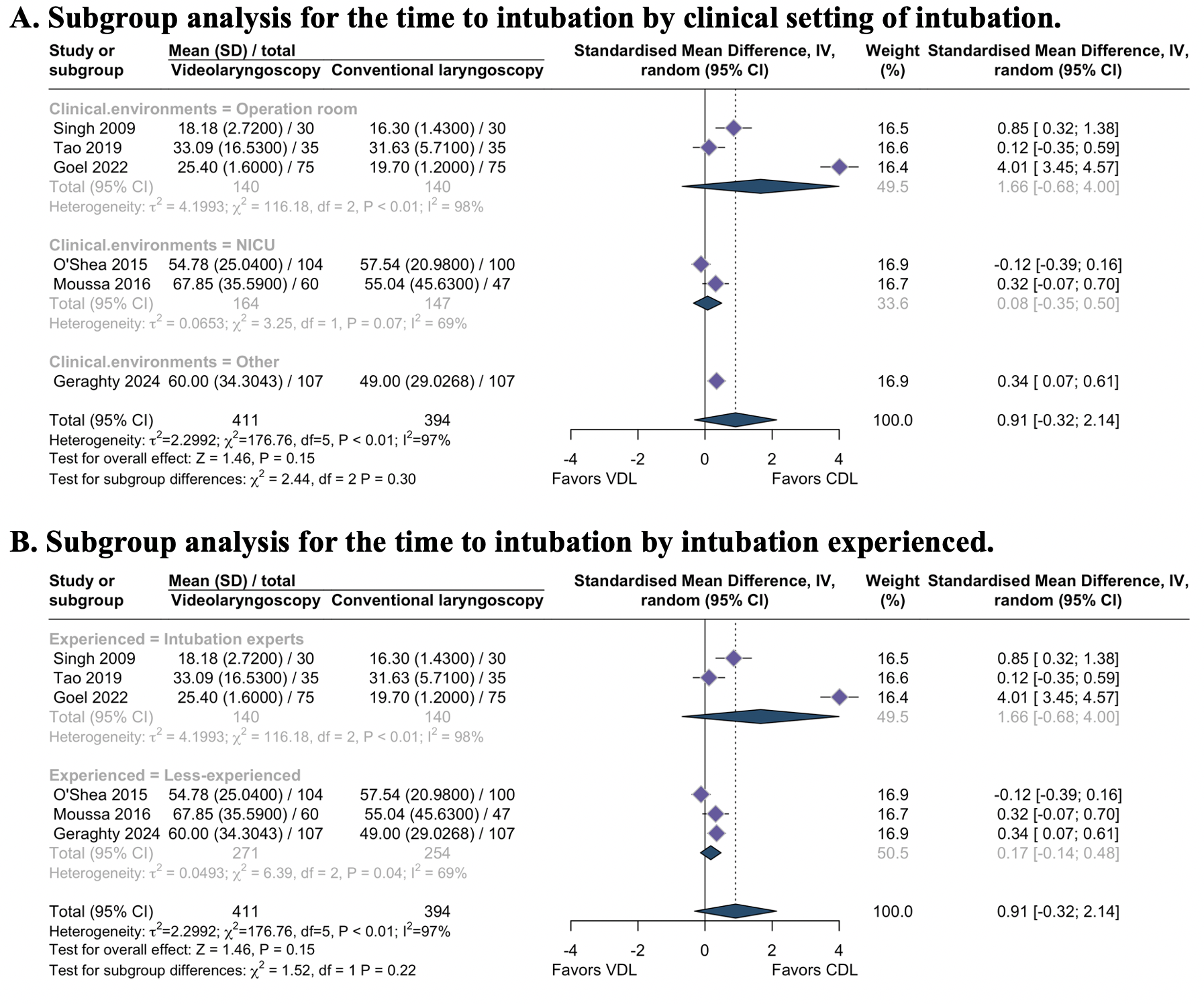
**eFigure 4. Subgroup analysis for** **time required for successful intubation.**

Abbreviation: CDL, conventional direct laryngoscopy; CI, confidence interval; VDL, Videolaryngoscopy.

**eFigure 5. Trial sequential analyses for number of intubation attempts.**


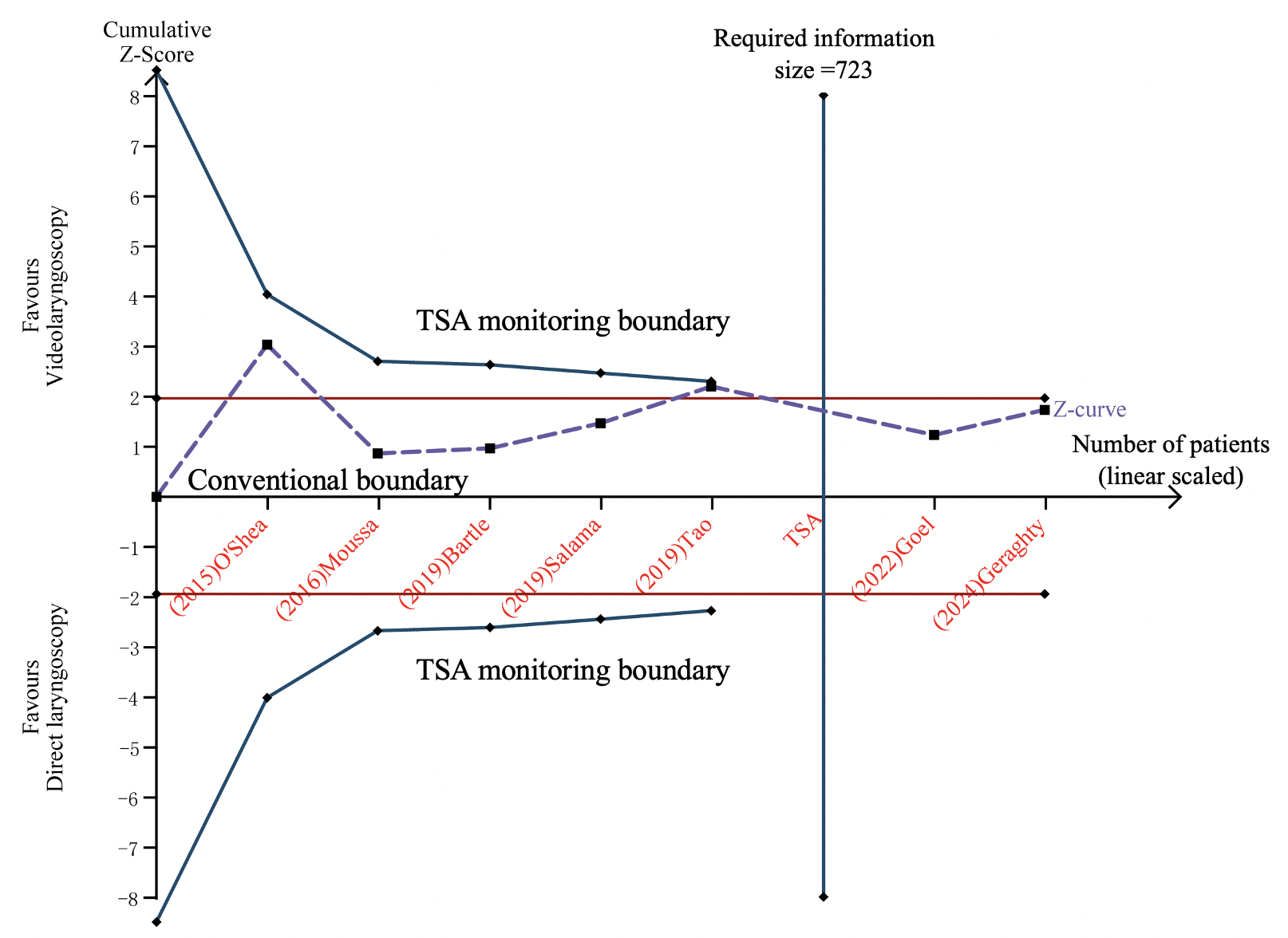


Abbreviation: TSA, Trial sequential analyses.


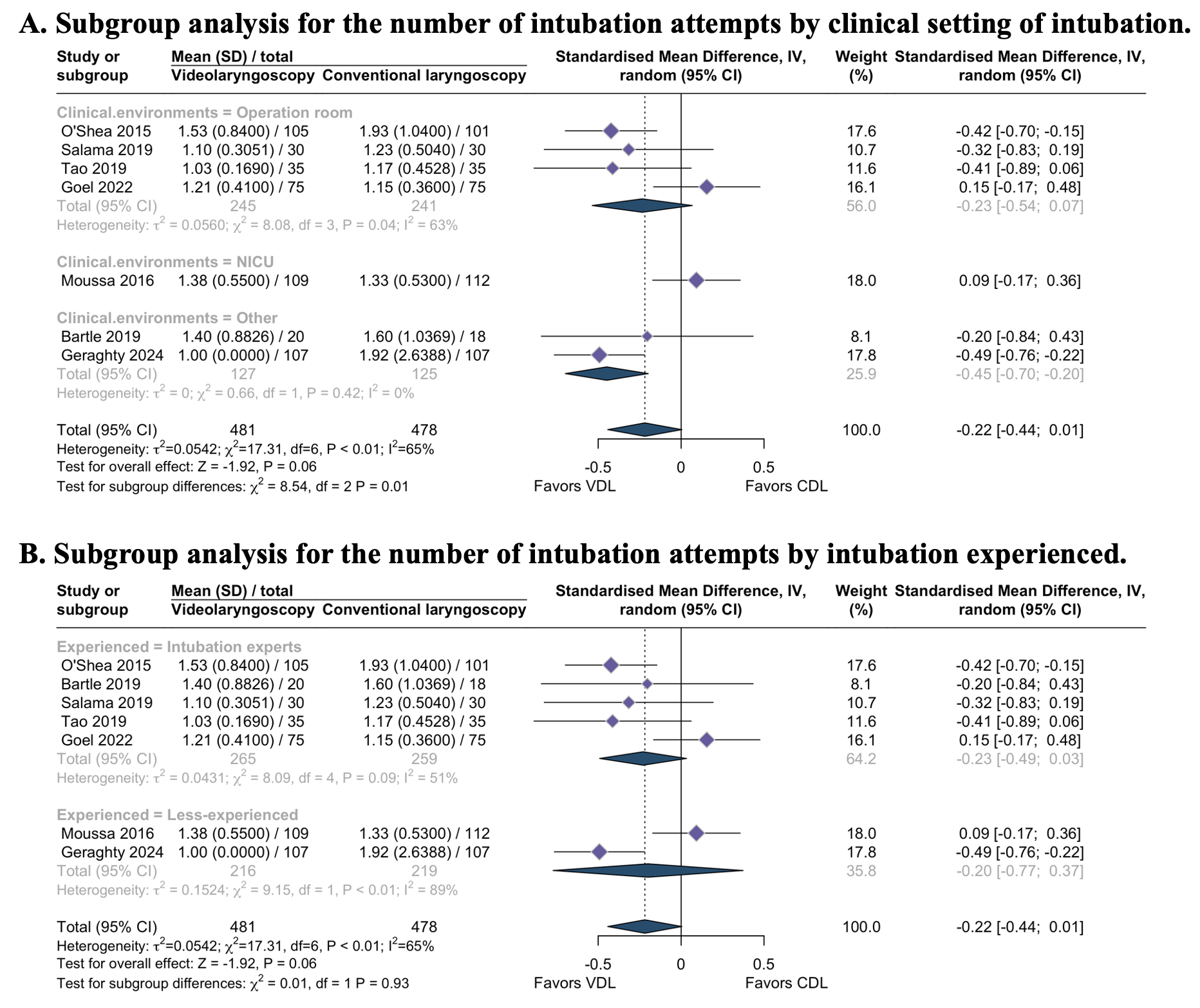
**eFigure 6. Subgroup analysis for** **number of intubation attempts.**

Abbreviation: CDL, conventional direct laryngoscopy; CI, confidence interval; VDL, Videolaryngoscopy.
